# Self-reported cancer in Brazil: data from the National Health Survey, 2013 and 2019

**DOI:** 10.11606/s1518-8787.2026060007291

**Published:** 2026-08-13

**Authors:** Ana Beatriz Machado de Almeida, Matheus Souza Ferreira, Gizelton Pereira Alencar, Tatiana Natasha Toporcov, Zilda Pereira da Silva

**Affiliations:** I Universidade de São Paulo Faculdade de Saúde Pública São Paulo SP Brasil Universidade de São Paulo. Faculdade de Saúde Pública. São Paulo, SP, Brasil

**Keywords:** Cancer, Prevalence, Health Surveys

## Abstract

**OBJECTIVE:**

To estimate the prevalence of self-reported cancer history in Brazil, describe the sociodemographic profile, identify the most common cancer types, and assess whether the condition limits individuals’ usual activities.

**METHODS:**

Descriptive study based on data from the Brazilian National Health Survey (PNS), conducted in 2013 and 2019. Data were obtained from the individual questionnaire, answered by a selected resident aged 18 or older. Self-reported cancer was defined by an affirmative response to the question: “Has a doctor ever given you a cancer diagnosis?” and the type of cancer was identified by the question: “At the time of your first cancer diagnosis, what type of cancer do/did you have?” Prevalence estimates and 95% confidence intervals were calculated. Analyses were performed using the R survey package (version 4.5.0), including the calibration process.

**RESULTS:**

In Brazil, 1.8% of individuals aged 18 or older (2.7 million adults) in 2013 and 2.4% (4.0 million adults) in 2019 reported having received a cancer diagnosis. The prevalence of self-reported cancer was higher among women in both years. Among men, prostate cancer was the most frequently reported, while among women, breast cancer was the most common in both periods. The average age at first cancer diagnosis was 51.8 years in 2013 and 50.5 years in 2019. Lung cancer was cited as the type that most limited individuals’ usual activities in both survey years.

**CONCLUSIONS:**

The PNS findings reveal a significant increase in the self-reported prevalence of cancer in Brazil. This growth may reflect advances in detection, greater public awareness, or changes in disease incidence. Despite the limitations inherent to the use of self-reported data, the representativeness of the sample allows for important generalizations to the Brazilian adult population.

## INTRODUCTION

Cancer is a disease of uncontrolled proliferation by transformed cells subject to evolution by natural selection^1^and represents a significant societal, public health, and economic challenge in the 21st century^2^. Globally, cancer is the second leading cause of death, and projections indicate that its burden will continue to grow over the next two decades^3^. This challenge is particularly pronounced in low- and middle-income countries, which account for 70% of the total cancer mortality^4^. In 2022, there were approximately 20 million new cases of cancer worldwide^2^, and in Brazil, an estimated 704,080 new cases are expected in 2025^5^. Furthermore, in 2023, cancer accounted for 17.4% of all deaths in Brazil, making it the second most common cause of mortality in the country, second only to cardiovascular diseases (CVD)^6^.

However, despite its current position, evidence from many high-income countries suggests an ongoing epidemiological transition in which cancer surpasses CVD mortality, highlighting the evolving dynamics of global and regional health challenges^7^. In Brazil, this trend is evident in data from 2000 to 2019, which show a notable decline in CVD mortality rates across 25 of the country’s 27 states, whereas cancer mortality rates increased in 15 states. In 2000, cancer mortality was lower than CVD mortality in all states, surpassing CVD in only 7% of municipalities. By 2019, however, this gap had narrowed significantly, with 13% of municipalities reporting higher cancer mortality rates compared to CVD^8^.

The increasing burden of cancer underscores the need for precise health impact quantification to guide targeted interventions and equitable access to prevention, detection, and treatment. While cancer registries are valid sources of data and a valuable tool for cancer surveillance and control in Brazil^9^, they can be administratively demanding and unfeasible, making self-reported disease history a cost- and time-effective approach for large-scale population-based research studies^10^.

The growing societal burden of cancer underscores the urgent need for precise quantification of its health impact. Accurate measurement is crucial for planning targeted interventions and ensuring equitable access to prevention, early detection, and effective treatment strategies. In this context, the present study aimed to estimate the prevalence of self-reported cancer in Brazil based on data from the *Pesquisa Nacional de Saúde* (PNS - National Health Survey) conducted in 2013 and 2019. Furthermore, the study aimed to describe the sociodemographic characteristics of people who reported a medical diagnosis of cancer, identify the most prevalent cancer types, evaluate whether cancer limits their usual activities, and compare these findings across the two survey years.

## METHODS

### Study Design

This was a descriptive study based on cross-sectional data from the PNS conducted in 2013 and 2019. The PNS is a population-based household survey conducted by the Instituto Brasileiro de Geografia e Estatística (IBGE - Brazilian Institute of Geography and Statistics)^a^ in partnership with the Ministry of Health.

### PNS Survey and Sample

The selection of participants was based on cluster probability sampling in three stages of selection and stratification of the primary sampling units. In the first stage, the selection of the primary sampling unit (census tracts or tract composition) was carried out by simple random sampling with the same stratification as the main sample. In the second stage, a fixed number of permanent private households was randomly selected in each primary sampling unit selected in the first stage from the National Register of Addresses for Statistical Purposes. In the third stage, a resident aged 18 years or older (2013) and 15 years or older (2019) was selected from a list of residents built at the time of the interview. For each of the three stages, a simple random sampling was performed for the selection of the units.

The PNS questionnaire is divided into three parts: (1) household information, (2) information about all household residents, and (3) individual information selected at random. Home-based questionnaires for all household residents are answered by a resident of the household who knows how to inform about the socioeconomic and health status of all residents. A selected resident answers the individual questionnaire with equiprobability among all adult household residents.

The IBGE was responsible for the fieldwork in both editions of the PNS. In 2013, 69,994 households were occupied, and 64,348 interviews were completed, yielding a non-response rate of 8.1%. By 2019, 108,525 households were visited, resulting in 94,114 interviews, with a non-response rate of 6.4% (Figure 1).

**Figure 1:**
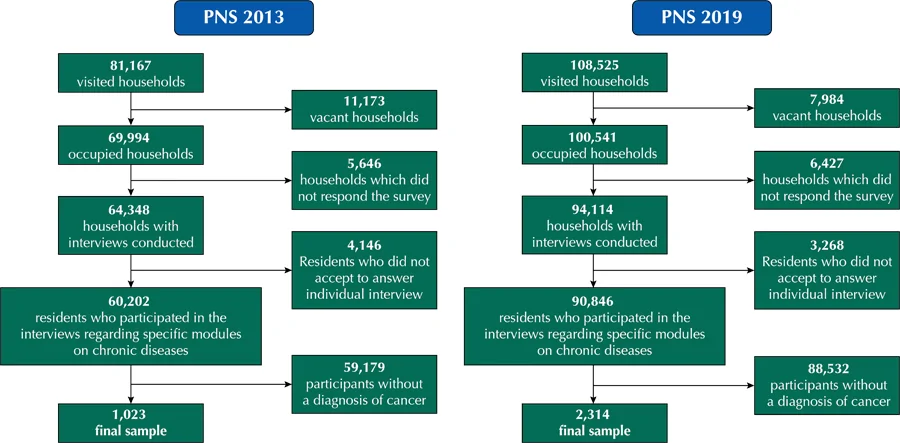
Flowchart of study participants, Brazilian National Health Survey, 2013 and 2019.

PNS: *Pesquisa Nacional de Saúde* (National Health Survey).

A calibration process based on population projections for Brazil and Federation Units by gender and age was carried out after weighing the bases by natural expansion factors. IBGE calibrated the expansion factors of the 2013 PNS to allow comparisons between the 2013 and 2019 editions of the PNS.

### Study Population

For the present study, data from the individual questionnaire—which included Module Q on chronic diseases—were used. This questionnaire was answered by a selected resident aged 18 years and older. Self-reported cancer was defined by a positive response to the question: “Has any doctor ever given you a cancer diagnosis?” (Question Q120 in both 2013 and 2019). Additionally, other self-reported cancer-related questions were analyzed within the subpopulation of adults who reported a cancer diagnosis:

*Type of cancer at the first diagnosis:* “At the first diagnosis of cancer, what type of cancer do you have or did you have?” (Question 121 in 2013); and “Was it a skin cancer diagnosis?”, “Was the diagnosed skin cancer of the melanoma type?”, and “Have you been diagnosed with another type of cancer?” (Questions Q121a, Q121b, and Q121c, respectively). In both years, the response options included: lung, intestine, stomach, breast (only for women), cervical cancer (only for women), prostate cancer (only for men), skin, and others.

*Age at the first cancer diagnosis:* “How old were you at the time of your first cancer diagnosis?” (Question 122 in 2013); and “How old were you at the time of your first cancer diagnosis (excluding non-melanoma skin cancer)?” (Question 122a in 2019).

*Presence and degree of disability:* “In general, to what extent does cancer or any problem caused by cancer limit your usual activities (such as working, doing household chores, etc.)?” (Question 123 in 2013); and “In general, does your treatment or any problem caused by cancer limit your professional or usual activities (such as working, doing household chores, etc.)?” (Question 123a in 2019). The response options were: “does not limit,” “a little,” “moderately,” “intensely,” and “very intensely.” For the purpose of this study, the responses were categorized as “not limit” and “limit,” with the latter grouping the categories “a little,” “moderately,” “intensely,” and “very intensely.”

Sociodemographic variables were derived from the PNS questionnaires and included sex (male and female), age (in years: 18–29, 30–49, 50–59, 60–64, 65–69, 70–74, and 75 or older), race/color (white, black, yellow [Asian], brown, or indigenous). Education level was categorized as follows: “incomplete elementary” (no formal education or incomplete elementary school), “complete elementary” (complete elementary school or incomplete high school), “complete high school” (complete high school or incomplete higher education), and “complete higher education.”” Additional variables included region (Mid-West, Northeast, North, Southeast, and South), and place of residence (urban or rural).

Data on private health insurance status (yes or no) were also included. In the PNS 2019, two separate questions addressed health insurance: “Do you have any private, company, or public agency dental insurance plan?” and “Do you have any private, company, or public agency medical insurance plan?” In contrast, the PNS 2013 featured a single question encompassing both types of coverage: “Do you have any private, company, or public agency health, medical, or dental plan?” Although an additional question specifically asked about dental plans — “Do you have a health plan just for dental care?”—this did not allow differentiation of respondents with exclusively medical coverage. As in previous studies, medical and dental coverage were jointly evaluated for comparisons between 2013 and 2019^11^.

### Statistical Analysis

In the statistical analysis, the PNS sampling design was accounted for, incorporating sample weights and addressing the clustering effect. All analyses were performed using the survey package in R statistical software (version 4.5.0), which accounts for weighting in complex sample designs. Within each survey edition, the association between cancer diagnosis and qualitative independent variables, as well as the association between the cancer site and qualitative independent variables, was tested using the Rao-Scott corrected chi-square test. The association between cancer site and age of first diagnosis was assessed via adjusted Wald tests derived from survey-weighted linear regression models.

### Ethical Aspects

The PNS received approval from the National Research Ethics Committee , ensuring that all research was conducted following ethical guidelines and regulations. The 2013 PNS was approved in July 2013 under number 328.159, and the 2019 PNS was approved in August 2019 under number 3.529.376. Participation in the survey by adults was voluntary, and confidentiality of information was guaranteed.

## RESULTS

The PNS estimated that 1.8% of individuals aged 18 and older (2.7 million adults) in 2013 and 2.4% (4.0 million adults) in 2019 reported a medical diagnosis of cancer in Brazil. The prevalence of self-reported cancer was higher among women in both years. Among males, it showed a slight increase, rising from 1.6% in 2013 to 2.0% in 2019. For females, the increase was more pronounced, from 2.1% in 2013 to 2.8% in 2019 (Table 1).

**Table 1:** Distribution and prevalence of self-reported cancer in adults (≥ 18 years) by sociodemographic variables. Brazil, 2013 and 2019.

Sociodemographic variables	PNS 2013		PNS 2019	
	% (95%CI)	Prevalence (95%CI)	% (95%CI)	Prevalence (95%CI)
Sex				
Male	40.6 (35.3– 46.2)	1.6 (1.3–1.9)	39.1 (35.8–42.5)	2.0 (1.8–2.2)
Female	59.4 (53.8–64.7)	2.1 (1.8–2.4)	60.9 (57.5–64.2)	2.8 (2.5–3.0)
Age (years)				
18–29 years	*	*	2.1 (1.3–3.4)	0.2 (0.2–0.4)
30–49 years	17.5 (13.7–22.1)	0.8 (0.6–1.1)	20.5 (17.7–23.5)	1.3 (1.1–1.6)
50–59 years	23.4 (19.5–27.9)	2.7 (2.2–3.2)	20.1 (17.4–23.1)	3.0 (2.6–3.5)
60–64 years	11.7 (8.6–15.7)	3.7 (2.7–5.1)	12.9 (10.7–15.4)	4.9 (4.1–5.9)
65–69 years	16.1 (12.6–20.3)	6.7 (5.2–8.5)	13.4 (11.2–16.0)	6.3 (5.2–7.6)
70–74 years	8.2 (6.0–11.2)	4.6 (3.3–6.3)	11.8 (9.9–14.0)	7.7 (6.4–9.2)
≥ 75 years	18.4 (14.4–23.1)	7.4 (5.9–9.4)	19.2 (16.8–21.9)	8.9 (7.8–10.1)
Race/Color				
White	67.3 (62.3–72.1)	2.6 (2.3–3.0)	61.2 (57.9–64.4)	3.4 (3.2–3.8)
Black	7.2 (5.0–10.1)	1.4 (1.0–2.1)	7.1 (5.7–8.9)	1.5 (1.2–1.9)
Brown	24.5 (20.4–29.0)	1.1 (0.9–1.3)	29.6 (26.6–32.7)	1.6 (1.4–1.8)
Yellow (Asian)	*	*	*	*
Indigenous	*	*	*	*
Education level				
Incomplete elementary	48.9 (43.5–54.4)	2.3 (2.0–2.7)	44.6 (41.2–48.1)	3.1 (2.9–3.5)
Complete elementary	9.3 (6.9–12.3)	1.1 (0.8–1.5)	10.9 (8.6–13.7)	1.5 (1.2–1.9)
Complete high school	20.8 (17.3–24.8)	1.2 (1.0–1.4)	24.2 (21.4–27.3)	1.8 (1.5–2.0)
Complete higher education	21.0 (16.5–26.4)	3.0 (2.4–3.9)	20.3 (17.7–23.2)	3.3 (2.9–3.8)
Region				
North	3.6 (2.6–5.0)	0.9 (0.7–1.2)	3.9 (3.2–4.7)	1.2 (1.0–1.4)
Northeast	13.4 (10.7–16.6)	0.9 (0.8–1.1)	18.2 (16.1–20.5)	1.7 (1.5–1.9)
Mid-West	6.8 (5.4–8.6)	1.7 (1.4–2.1)	7.1 (5.9–8.5)	2.2 (1.9–2.7)
South	25.4 (20.8–30.6)	3.2 (2.6–3.9)	20.3 (18.0–23.0)	3.4 (3.0–3.8)
Southeast	50.8 (45.1–56.5)	2.1 (1.8–2.5)	50.5 (47.0–53.9)	2.8 (2.5–3.2)
Place of residence				
Urban	91.1 (87.9–93.6)	1.9 (1.7–2.2)	90.6 (89.1–92.0)	2.5 (2.4–2.7)
Rural	8.9 (6.4–12.1)	1.2 (0.9–1.5)	9.4 (8.0–10.9)	1.6 (1.4–1.9)
Private health insurance				
Yes	44.3 (38.8–50.0)	2.7 (2.3–3.2)	41.8 (38.2–45.4)	3.5 (3.1–3.9)
No	55.7 (50.0–61.2)	1.5 (1.3–1.7)	58.2 (54.6–61.8)	2.0 (1.8–2.2)

PNS: *Pesquisa Nacional de Saúde* (National Health Survey); IBGE: *Instituto Brasileiro de Geografia e Estatística* (Brazilian Institute of Geography and Statistics); 95%CI: 95% confidence interval.

*Values that the corresponding coefficients of variation exceeded 30%, the threshold established by IBGE to identify values that could not be reliably estimated.

Cancer prevalence increased with aging in both surveys. The youngest age group (18–29 years) recorded the lowest prevalence (0.2%) of self-reported cancer in 2019. While most age groups showed an increase in prevalence over the years, the 65–69 age group experienced a slight decline, from 6.7% in 2013 to 6.3% in 2019. In contrast, older age groups exhibited significantly higher prevalence rates, particularly those aged 75 years and older, whose prevalence rose from 7.4% in 2013 to 8.9% in 2019. People who self-declared race/color white had the highest prevalence rates, increasing from 2.6% in 2013 to 3.4% in 2019. The black and brown populations also experienced slight increases, with rates rising from 1.4% to 1.5% and from 1.1% to 1.6%, respectively (Table 1).

All education levels showed an increase in prevalence rates over the years. Individuals with complete higher education consistently exhibited the highest rates, rising from 3.0% in 2013 to 3.3% in 2019. The most notable increase was observed among those with incomplete elementary education, with rates ranging from 2.3% in 2013 to 3.1% in 2019 (Table 1).

Considering the place of residence, all regions showed an increase in cancer prevalence over time. The South Region recorded the highest prevalence of self-reported cancer in both periods, with 3.2% in 2013 and 3.4% in 2019. In contrast, the North and Northeast Regions reported the lowest prevalence rates in 2013 and 2019, both starting at 0.9% in 2013 and increasing to 1.2% and 1.7%, respectively, in 2019. Both places of residence showed an increase in prevalence between the periods, with urban areas exhibiting significantly higher rates than rural areas. In urban areas, prevalence increased from 1.9% in 2013 to 2.5% in 2019. Rural areas, while experiencing a smaller increase, also showed a rise in prevalence from 1.2% to 1.6% (Table 1).

Individuals with private health insurance exhibited significantly higher prevalence rates than those without insurance. Among those with private coverage, prevalence increased from 2.7% in 2013 to 3.5% in 2019. Similarly, individuals without insurance also showed an increase, rising from 1.5% to 2.0% during the same period (Table 1).

At a 5% significance level, all analyzed sociodemographic variables were statistically significantly associated with self-reported cancer diagnosis in both 2013 and 2019.

Regarding the types of cancer diagnosed, prostate cancer was the most frequently reported among men in both periods—15.6% (95%CI: 12.0–20.0) in 2013 and 13.8% (95%CI: 11.7–16.3) in 2019—followed by skin cancer, with rates of 7.8% (95%CI: 5.2–11.3) in 2013 and 9.3% (95%CI: 7.6–11.3) in 2019, and intestinal cancer, with 4.0% (95%CI: 2.0–7.8) in 2013 and 2.0% (95%CI: 1.4–2.8) in 2019 (Figure 2).

**Figure 2:**
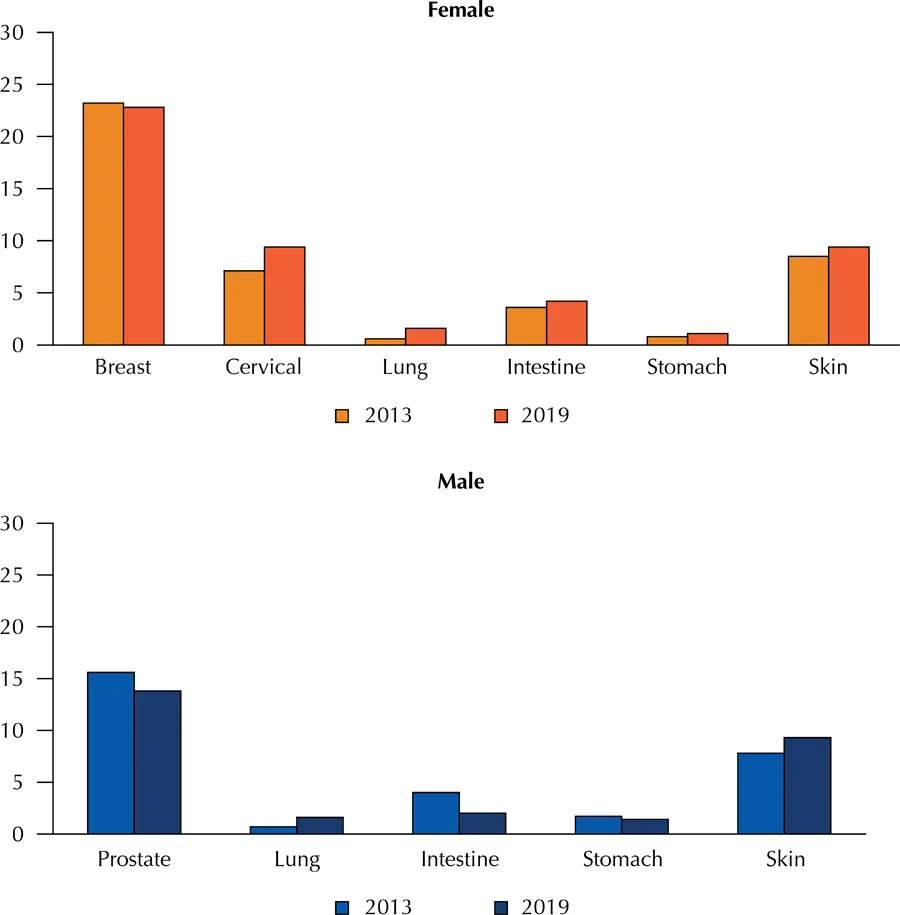
Distribution of adults who self-reported cancer by site according to sex. Brazil, 2013 and 2019.

Among women, breast cancer remained the most frequently reported diagnosis in both periods—23.2% (95%CI: 18.7–28.6) in 2013 and 22.8% (95%CI: 19.8–26.2) in 2019—followed by skin cancer, with rates of 8.5% (95%CI: 6.4–11.3) in 2013 and 9.4% (95%CI: 7.5–11.6) in 2019, and cervical cancer, with 7.1% (95%CI: 4.8–10.3) in 2013 and 9.4% (95%CI: 7.7–11.5) in 2019 (Figure 2).

The mean age of first cancer diagnosis was 51.8 years (95%CI: 49.9–53.7) in 2013 and 50.5 years (95%CI: 49.3–51.7) in 2019. The lowest mean age was observed for cervical cancer in both periods (35.4 years and 41.8 years, respectively), while the highest was reported for prostate cancer (65.7 years in 2013 and 62.9 years in 2019) (Table 2).

**Table 2:** Distribution of mean age of the first cancer diagnosis among adults who self-reported cancer. Brazil, 2013 and 2019.

Location	PNS 2013	PNS 2019
	Mean age (95%CI)	Mean age (95%CI)
Breast (female)	48.8 (45.0–52.5)	48.7 (46.2–51.2)
Cervical (female)	35.4 (29.8–41.1)	41.8 (38.9–44.7)
Prostate (male)	65.7 (63.9–67.4)	62.9 (60.8–65.0)
Lung		
Male	63.9 (51.9–76.0)	55.5 (47.6–63.3)
Female	51.0 (43.8–58.3)	51.8 (43.2–60.5)
Both sexes	57.8 (49.0–66.6)	53.7 (48.0–59.4)
Intestine		
Male	56.4 (48.0–64.7)	55.1 (49.3–60.9)
Female	59.7 (52.5–66.8)	51.5 (45.4–57.7)
Both sexes	57.9 (52.4–63.5)	52.7 (48.1–57.3)
Stomach		
Male	47.6 (39.3–56.0)	59.0 (54.2–63.8)
Female	53.3 (40.6–66.0)	53.6 (45.2–62.0)
Both sexes	49.5 (42.1–56.9)	56.6 (52.1–61.1)
Skin		
Male	54.7 (44.6–64.8)	55.1 (49.9–60.3)
Female	50.8 (45.9–55.8)	52.8 (49.1–56.6)
Both sexes	52.7 (47.0–58.3)	54.1 (50.7–57.5)
Other cancers		
Male	51.6 (46.2–57.0)	48.1 (44.6–51.5)
Female	45.9 (41.0–50.8)	43.2 (40.2–46.2)
Both sexes	48.2 (44.4–52.1)	45.5 (43.1–47.8)

PNS: *Pesquisa Nacional de Saúde* (National Health Survey); 95%CI: 95% confidence interval.

When comparing the two PNS periods, a decrease in the mean age at diagnosis was observed for several cancers. For breast cancer, the mean age dropped slightly from 48.8 years in 2013 to 48.7 years in 2019. For lung cancer among males, the age decreased significantly from 63.9 to 55.5 years. Intestine cancer also showed a decline in mean age for both sexes, with male patients decreasing from 56.4 to 55.1 years and female patients from 59.7 to 51.5 years. Other types of cancers showed a reduction in mean age from 48.2 years to 45.5 years (Table 2).

In contrast, an increase in mean age was noted for stomach cancer, which rose from 49.5 years in 2013 to 56.6 years in 2019, driven mainly by an increase among males (from 47.6 years to 59.0 years). Similarly, the mean age at diagnosis for skin cancer increased from 52.7 years to 54.1 years for both sexes, with a notable rise among females (from 50.8 years to 52.8 years) (Table 2). There is no statistically significant evidence of a difference in mean age at diagnosis among the considered cancer type groups.

Analyzing the presence and degree of disability by cancer type, lung cancer was reported as the most limiting to individuals’ usual activities in both survey periods. In 2013, 85.0% of respondents indicated that lung cancer restricted their daily routines, while in 2019, this figure decreased to 61.8%. When analyzing by sex, women reported higher levels of disability associated with lung cancer: 97.9% in 2013 compared to 73.2% in men. In 2019, this trend continued, with 67.5% of women reporting limitations versus 56.4% of men (Table 3).

**Table 3:** Distribution of adults who self-reported that cancer limits their usual activities. Brazil, 2013 and 2019.

Location	PNS 2013	PNS 2019
	% (95%CI)	% (95%CI)
Breast	32.6 (23.1–43.9)	33.5 (26.6–41.1)
Cervical	9.9 (4.4–20.6)	19.7 (12.2–30.2)
Prostate	36.6 (22.9–52.8)	31.1 (22.6–41.2)
Lung		
Male	73.2 (^a^)	56.4 (12.7–92.0)
Female	97.9 (^a^)	67.5 (24.3–93.1)
Both sexes	85.0 (^a^)	61.8 (39.9–79.8)
Intestine		
Male	25.8 (1.8–87.0)	35.7 (17.3–59.6)
Female	52.6 (24.7–78.9)	39.5 (23.5–58.1)
Both sexes	38.5 (19.8–61.4)	38.3 (26.4–51.8)
Stomach		
Male	34.1 (1.8–93.7)	42.9 (7.7–87.1)
Female	14.9 (0.0–^a^)	43.0 (14.1–77.6)
Both sexes	27.8 (7.4–64.9)	42.9 (22.6–65.9)
Skin		
Male	32.6 (16.1–54.8)	21.3 (10.9–37.4)
Female	32.6 (18.2–51.1)	11.3 (5.5–21.9)
Both sexes	32.6 (21.6–45.8)	17.0 (10.4–26.5)
Other cancers		
Male	37.9 (23.4–55.0)	37.4 (27.7–48.3)
Female	38.3 (26.0–52.3)	24.9 (17.6–34.0)
Both sexes	38.1 (28.6–48.6)	30.7 (24.5–37.7)

PNS: *Pesquisa Nacional de Saúde* (National Health Survey); 95%CI: 95% confidence interval.

^a^ These estimates had a coefficient of variation greater than 30%, indicating limited precision.

The analysis revealed an increase in the proportion of women reporting limitations in daily activities due to cancer diagnoses between the years 2013 and 2019. Breast cancer showed a slight variation, rising from 32.6% in 2013 to 33.5% in 2019. In contrast, cervical cancer exhibited a substantial increase, doubling the reported rate of activity limitation from 9.9% to 19.7%. Stomach cancer stood out with the greatest change recorded, increasing from 14.9% to 43.0% (Table 3).

Among men, there was a notable increase in the proportion of individuals reporting limitations in daily activities due to cancer diagnoses, particularly for intestinal and stomach cancers, between 2013 and 2019. The percentage of men reporting functional limitations due to intestinal cancer rose from 25.8% in 2013 to 35.7% in 2019, while reported limitations from stomach cancer increased from 34.1% to 42.9% over the same period (Table 3).

The analysis revealed a statistically significant association between limitations in daily activities and self-reported cancer diagnosis in both 2013 (p = 0.0148) and 2019 (p = 0.0002), considering a 5% significance level.

## DISCUSSION

The findings from the PNS indicate a significant increase in the self-reported prevalence of cancer in Brazil between 2013 and 2019. This may reflect advances in detection, increased public awareness, or real changes in disease incidence. The rise was more pronounced among women, which could be associated with a higher frequency of preventive screenings, longer life expectancy, or the prominence of highly visible cancer types such as breast and cervical cancer.

The association between aging and increased cancer prevalence aligns with global literature, highlighting age as a key risk factor. Older age groups consistently presented higher rates, particularly those aged 75 and above. However, the increase observed among individuals with lower education levels and those without health insurance suggests structural inequalities in access to early diagnosis and treatment, requiring attention from public health policies.

The analysis of the most frequently diagnosed cancers confirmed established patterns, such as the predominance of prostate cancer among men and breast cancer among women. Nevertheless, the data on functional limitations reveal less commonly explored dimensions: certain cancer types, such as lung, stomach, and intestinal cancers, showed high levels of impairment in daily activities—especially among women, possibly reflecting differences in symptom perception, disease severity, received social support, or gender aspects.

Additionally, the observed reduction in the mean age at diagnosis for certain cancers, such as lung and intestinal cancers, suggests shifts in population risk factors or improved screening strategies. Conversely, the increase in mean age at diagnosis for stomach cancer may indicate delays in symptom recognition or access to specialized care.

The substantial increase in the proportion of women reporting limitations due to cervical and stomach cancer deserves special attention. These findings may point to persistent barriers in accessing primary care, early diagnosis, and psychosocial support. Similarly, among men, a concerning trend emerged regarding the growing functional impact of gastric and intestinal cancers.

Potential limitations of our study should be mentioned. Cancer was defined by self-report, which is a cost- and time-effective approach for large-scale population-based research studies. However, self-reports may introduce recall bias or misclassification^12^, potentially leading to an underestimation of total prevalence^13^.

Although the core question used to define self-reported cancer was identical in both surveys, some differences in questionnaire structure between 2013 and 2019 should be considered when interpreting temporal comparisons. In 2019, additional detail was introduced for skin cancer (including melanoma classification), and the question on age at diagnosis explicitly excluded non-melanoma skin cancer, which may affect comparability with 2013 estimates. Moreover, the wording of the activity limitation question differed slightly, incorporating treatment-related impacts and professional activities in 2019, which may have affected comparability. Differences in the measurement of private health insurance coverage may also have influenced subgroup comparisons. These variations may introduce measurement bias and should be considered when evaluating trends over time. However, the use of identical core definitions for cancer diagnosis and the calibration of sampling weights by IBGE to ensure comparability between editions strengthen the validity of the observed patterns.

One of the main strengths of this analysis is the representative nature of the data, allowing the results to be generalized to the Brazilian adult population. The evaluation of self-reported data highlights the burden of cancer in Brazil and contributes to the identification of disparities in diagnosis, access to healthcare, and disease management. Moreover, population-based surveys, such as the PNS, are essential tools for cancer surveillance, complementing traditional information systems by capturing self-reported diagnoses, social inequalities, and the functional impact of the disease.

These findings reinforce the importance of maintaining periodic national surveys to monitor trends over time and to support the planning and evaluation of public health policies aimed at improving cancer detection, reducing inequities, and enhancing quality of life.

## Footnotes

a The database is available from: https://www.ibge.gov.br/estatisticas/sociais/saude/9160-pesquisa-nacional-de-saude.html?=&t=microdados
